# Mitonuclear interactions, mtDNA-mediated thermal plasticity, and implications for the Trojan Female Technique for pest control

**DOI:** 10.1038/srep30016

**Published:** 2016-07-21

**Authors:** Jonci N. Wolff, Daniel M. Tompkins, Neil J. Gemmell, Damian K. Dowling

**Affiliations:** 1 School of Biological Sciences, Monash University, Victoria, 3800, Australia; 2 Landcare Research, Private Bag 1930, Dunedin, New Zealand; 3 Allan Wilson Centre for Molecular Ecology and Evolution, Department of Anatomy, University of Otago, Dunedin 9016, New Zealand

## Abstract

Pest species pose major challenges to global economies, ecosystems, and health. Unfortunately, most conventional approaches to pest control remain costly, and temporary in effect. As such, a heritable variant of the Sterile Insect Technique (SIT) was proposed, based on the introduction of mitochondrial DNA mutations into pest populations, which impair male fertility but have no effects on females. Evidence for this “Trojan Female Technique” (TFT) was recently provided, in the form of a mutation in the mitochondrial cytochrome b gene (*mt:Cyt-b*) of *Drosophila melanogaster* which reduces male fertility across diverse nuclear backgrounds. However, recent studies have shown that the magnitude of mitochondrial genetic effects on the phenotype can vary greatly across environments, with mtDNA polymorphisms commonly entwined in genotype-by-environment (G × E) interactions. Here we test whether the male-sterilizing effects previously associated with the *mt:Cyt-b* mutation are consistent across three thermal and three nuclear genomic contexts. The effects of this mutation were indeed moderated by the nuclear background and thermal environment, but crucially the fertility of males carrying the mutation was invariably reduced relative to controls. This mutation thus constitutes a promising candidate for the further development of the TFT.

Pest species pose a significant threat to native biota, and cause major damage to natural environments and economies globally^1^. Conventional approaches to their control or eradication (for example through poisoning or trapping) can be laborious to implement, economically costly, temporary in nature, and cause undesirable environmental contamination, or detrimental effects on non-target species^2^,^3^,^4^. Much attention has thus focused on the development of control measures that are species-specific and long-lasting in effect, even when applied to pest populations at low density. One approach that meets the criteria of species-specificity is the sterile insect technique (SIT), whereby sterile males are introduced *en masse* into target populations, reducing offspring production of females that mate with them^5^. However, the SIT generally requires continuous large-scale production and introduction of sterile males to sustain population suppression^5^; it would be further improved if sterile males could be produced continually within the populations targeted for control, without requiring manual introductions each generation.

Significant research effort has thus been directed at developing novel SIT variants that promise trans-generational heritability of male sterility, and that ideally require only periodic releases of sterile males to successfully control pest populations^5^. Such approaches usually employ some form of genome editing to achieve both heritability and reproductive suppression. One such approach is the Release of Insects carrying a Dominant Lethal (RIDL™), whereby transgenic insects homozygous for a dominant repressible lethal gene are introduced into target populations^6^. A repressor for the lethal gene, *e.g*. a tetracycline-repressible transcription factor, allows for mass-breeding of insects reared on repressor-containing diets. Once released, and in the absence of the repressor, expression of the lethal gene is triggered by the expression of a female-specific transcription enhancer, killing females during development while leaving males unscathed. The survival of males enables trans-generational heritability, and feasibility of this repressible approach has been demonstrated in laboratory fruit fly populations^6^,^7^ and field mosquito populations^8^.

A challenge to SIT variants that remains is to establish effector genes at sufficiently high frequencies within target populations to achieve sustained control. An interesting and alternative approach to SIT, to help the spread of effector genes through target populations, is to cause their biased inheritance through linkage to site-specific selfish genetic elements (“gene drive”)^9^,^10^. This concept is not new, but has recently gained considerable traction with the development of the CRISPR-Cas9 (clustered, regularly interspaced, short palindromic repeats (CRISPR) and CRISPR-associated protein [Cas] system) genome editing technology^11^,^12^. Developing and applying a CRISPR-Cas9-based gene drive system, researchers have demonstrated effector gene transmission rates to offspring of over 90% in mosquitoes^11^. However, while promising very efficient means to control pest populations or disease vectors^11^,^13^, the release of genetically engineered animals into nature raises ethical issues, and a debate is currently underway discussing safety and regulatory concerns^14^,^15^.

A novel variant of the SIT, which in theory can achieve sustained population suppression over multiple generations in any taxa, and which does not rely on the introduction of novel gene constructs into nature, is the recently proposed Trojan Female Technique (TFT)^16^. The TFT is based on the use of naturally-occurring mutations in the mtDNA that incur reductions to male fertility while having no deleterious effects on females^17^,^18^. Males that inherit these mutations will sire fewer offspring than wild-type counterparts, while females will remain fully fertile. Since mtDNA is generally strictly maternally inherited, this sex-bias in effects means that under most conditions there will be greatly reduced selection pressure against the TFT mutation. Thus a single release of females carrying the TFT mutation into a pest population could potentially cause multi-generational population suppression^16^.

The applicability of the TFT to pest control has been theoretically substantiated^16^, but its empirical success, and ultimate practicability, hinges on circumvention of several potential obstacles. First, the effects of any given candidate TFT mutation on male fertility must be upheld across a range of nuclear genetic backgrounds. This is salient given previous findings indicating that the effects of polymorphisms within the mtDNA sequence are typically moderated by genetic variation in the nuclear genome^19^,^20^. That is, mtDNA haplotypes that might be poorly performing against one nuclear genomic background can often be high performers against others^21^,^22^,^23^. Mitonuclear interactions thus threaten to impede the development of the TFT. However, it has recently been shown that a non-synonymous candidate mutation in the mitochondrial genome of the fruit fly *Drosophila melanogaster (D. melanogaster*) indeed consistently impairs male fertility across a range of diverse nuclear genomic backgrounds^24^. The non-synonymous mutation causes a change from Alanine to Threonine at amino acid position 278 of the expressed protein of the mitochondrial cytochrome b gene (*mt:Cyt-b*), a component of the mitochondrial respiratory complex III^25^,^26^. Spermatogenesis of male fruit flies carrying the BRO haplotype is perturbed, inhibiting sperm maturation, which can lead to complete sterility while having no apparent effects on female fertility^26^. Although the detrimental effect of the *mt:Cyt-b* mutation on male fertility in *D. melanogaster* is well established^24^,^27^, the underlying mechanism that leads to the suppression of male fertility is yet to be resolved^26^. However, the potential for use of the *mt:Cyt-b* mutation for the TFT is particularly interesting given that the same mutation has already been identified in the *mt:Cyt-b* gene of a number of other species, both vertebrate and invertebrate^26^.

However, a second obstacle that could plausibly stand in the way of further development of the TFT is the scope for genotype-by-environment (GxE) interactions involving sequence polymorphisms in the mtDNA that alter male-fertility phenotypes (cases of “mtDNA-mediated phenotypic plasticity”). Evidence for such plasticity has accumulated in recent years. In early studies that examined mtDNA transmission patterns following experimentally-enforced heteroplasmy in *Drosophila*, observed patterns were not only shaped by the nuclear background but also by the temperature at which flies were reared^28^,^29^. Furthermore, in seed beetles, the effect of the mitochondrial haplotype on the speed of juvenile development and metabolic rate is contingent not only on the nuclear background, but also on complex interactions involving the mtDNA, nuclear background and thermal environment^30^,^31^. Since natural populations invariably exist within heterogeneous environments, and simultaneously face ongoing exposure to climate change, it is thus crucial to screen the effects of candidate TFT mutations across different thermal contexts to assess ongoing practicability. Here we do so for the described Ala278→Thr *mt:Cyt-b* mutation, assessing whether its fertility outcome for males is modified by interactions with the thermal environment. Because of the importance of the mitonuclear interaction in life-history expression, we further test the thermal interaction across three different non-coevolved nuclear backgrounds.

## Results and Discussion

**Figure 1:**
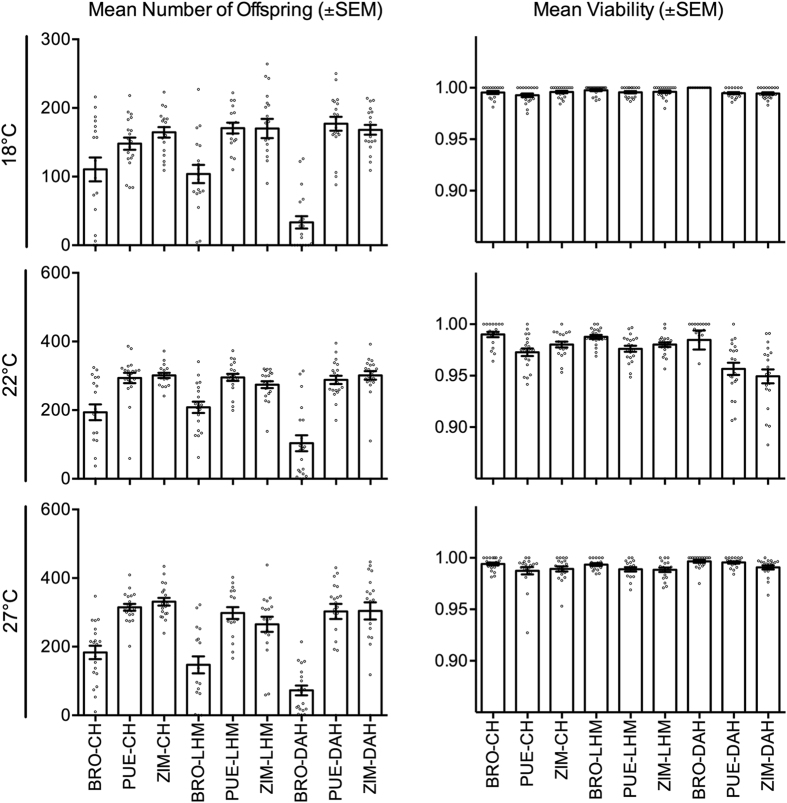
Mean offspring number and mean pupal viability per nuclear and thermal environment (±SEM) of three mitochondrial haplotypes (Brownsville [BRO]; Puerto Montt [PUE]; Zimbabwe [ZIM]) across three nuclear backgrounds (Coffs Harbour [CH], Dahomey [DAH], LHM [LHM]), and three thermal environments (18 °C, 22 °C, 27 °C).

**Figure 2:**
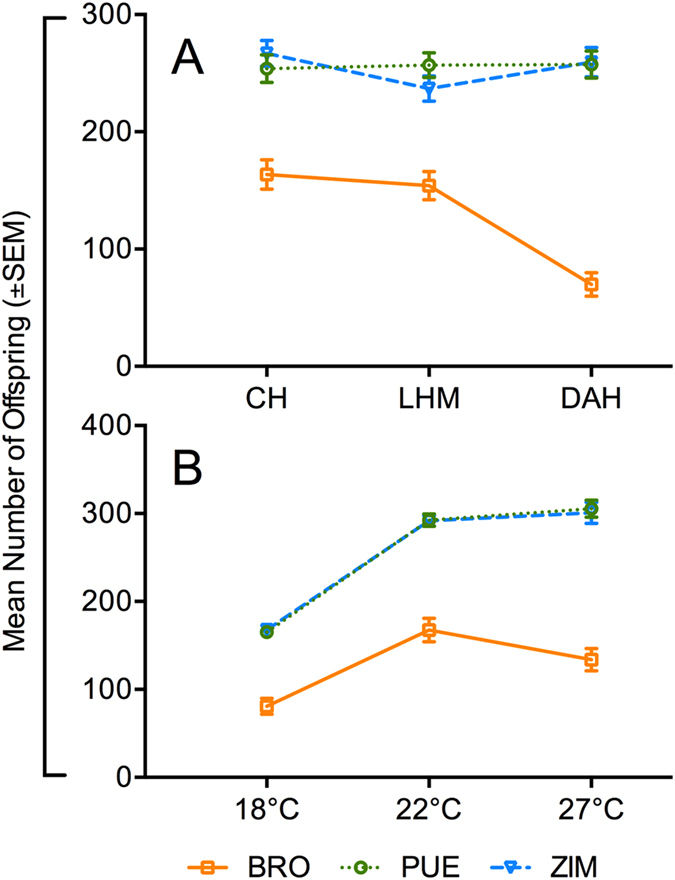
Mean offspring number per nuclear and thermal environment. Interaction plots depicting mean offspring number (±SEM) of three mitochondrial haplotypes (Brownsville [BRO]; Puerto Montt [PUE]; Zimbabwe [ZIM]) across (**A**) three nuclear backgrounds (Coffs Harbour [CH], Dahomey [DAH], LHM [LHM]), and (**B**) three thermal environments (18 °C, 22 °C, 27 °C).

To test whether the male-sterilizing effects previously associated with the *mt:Cyt-b* mutation are consistent across varying thermal and nuclear genomic contexts, we placed three different mtDNA haplotypes (one which harbours the candidate TFT mutation in the *mt:Cyt-b* gene [BRO] and two control haplotypes [PUE; ZIM]) alongside three foreign non-coevolved nuclear backgrounds (CH, DAH, or LHM) and measured their effects on male fertility at three different temperatures (for details see Methods; Fig. S1). We then conducted male fertility assays for all nine mitonuclear combinations, and determined offspring number and pupal viability (Fig. 1). We found context-dependent phenotypic effects of mtDNA mutations that hinged on both the nuclear background in which they were expressed (χ^2^ = 36.6, p < 0.001, Table 1) and the temperature at which they had been reared for three generations (χ^2^ = 24.9, p < 0.001, Table 1). Crucially, the average fertility of males with the BRO mtDNA haplotype, which carries the candidate TFT mutation, was consistently lower across all nuclear genomic and thermal contexts than that of males with either of the two control haplotypes (Figs 1 and 2). On average, the BRO haplotype conferred an approximate 50% reduction in fertility. Interestingly, the observed mitochondrially-mediated thermal plasticity was directly tied to the BRO haplotype; male fertility for the control haplotypes increased with assay temperature, whereas BRO-linked fertility peaked mid-temperature (22 °C; Figs 1 and 2).

The BRO haplotype was also the mediator of the mitonuclear interactions detected in this study. While male fertility of the two control haplotypes was similar across the three nuclear backgrounds, BRO-linked fertility was much reduced when expressed alongside the DAH nuclear background (reduced by ~72%) relative to CH or LHM (reduced by ~37% for both; Figs 1 and 2). Notably, none of three tested nuclear backgrounds, nor the *w*^*1118*^ nuclear background (alongside which male-sterilizing effects were first observed), have coevolved with the BRO haplotype. This indicates that variably successful mechanisms coded for by the nuclear genome (dependent on both the nuclear background and thermal conditions) already exist within the gene pools of tested nuclear backgrounds, and which may be acting to compensate for mtDNA-linked reductions in male fertility. The existence of such modifying mechanisms within the nuclear background is evolutionary plausible, because they would have been selected for over time by the natural occurrence of male fertility-reducing mtDNA variation in the wild populations^19^,^32^. Importantly, however, we note that despite the BRO haplotype being entwined in gene-by-gene (mitonuclear) and gene-by-environment (mito-by-temperature) interactions, it was invariably the inferior performer relative to the controls, reinforcing the promise of harnessing mtDNA mutations, such as the Ala278→Thr in the *mt:Cyt-b* gene, in the development of the TFT to aide eradication of pest species.

**Figure 3:**
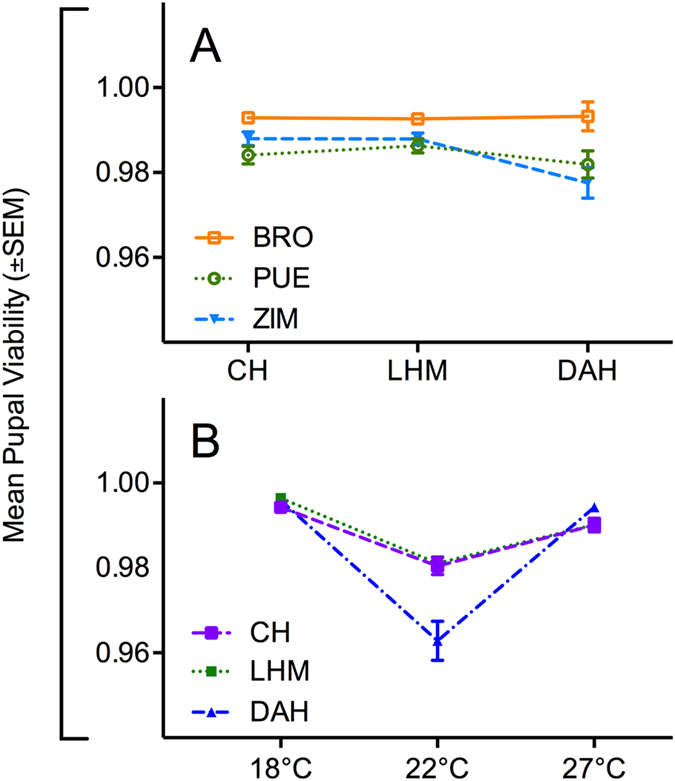
Mean pupal viability per nuclear and thermal environment. Interaction plots depicting mean pupal viability (±SEM) of three mitochondrial haplotypes (Brownsville [BRO], Puerto Montt [PUE], Zimbabwe [ZIM]) across (**A**) three nuclear backgrounds (Coffs Harbour [CH], Dahomey [DAH], LHM [LHM]), and (**B**) three nuclear backgrounds (CH, DAH, LHM) across three temperatures (18  °C, 22  °C, 27 °C).

Our analyses of pupal viability showed that while mitonuclear interactions affect viability rates (χ^2^ = 17.4, p = 0.0011, Table S2), invariably the BRO haplotype, which harbours the candidate TFT mutation, exhibited highest pupal viability (Figs 1 and 3). This indicates that the low male fertility associated with the BRO haplotype is not underpinned by low rates of pupal-to-adult eclosion. It is conceivable that higher pupal viability could aid the BRO haplotype to spread slowly within introduced populations following TFT treatment. However, further empirical testing is required to validate or refute this hypothesis. One approach would be to monitor changes in haplotype frequencies in mixed population cage studies, where the BRO haplotype is put in direct competition to other mtDNA haplotypes. Pupal viability was also affected by an interaction between the nuclear background and temperature (χ^2^ = 37.7, p < 0.001; Figs 1 and 3; Table S2). While pupal viability was lowest for all nuclear genotypes at 22 °C, this was particularly evident for the DAH background.

Together, our findings demonstrate that the thermal environment can play a role in male fertility outcomes of the Ala278→Thr *mt:Cyt-b* mutation in fruit fly. The observed mtDNA-mediated thermal plasticity for male fertility is a novel finding, and shows that trait expression reliant on genes encoded within the evolutionary-conserved mitochondrial genome is modified across environments. The underlying mechanisms remain to be resolved, but it is plausible that haplotype-specific changes in mitonuclear protein complexes alter efficiencies across environments of what are temperature-sensitive processes in the mitochondrial electron transport chain. Alternatively, it is also possible that observed thermal plasticity for male fertility is driven by changes in gene expression in response to mitochondrial genomic variation and temperature, both of which are known to alter gene expression^33^,^34^.

While it is possible that the context-dependency of the BRO haplotype might be traced directly to the Ala278→Thr *mt:Cyt-b* mutation, this requires further testing given that the BRO and control haplotypes are delineated by additional SNPs within the coding and non-coding region^25^. Such tests require placement of this candidate mutation inside other mitochondrial haplotype backgrounds, to disentangle direct effects of the SNP from that of the hosting haplotype. Also, the general applicability across species of this mutation for use as the basis for TFT pest control has yet to be demonstrated; the effects of this mutation must be tested in other taxa, most importantly those that represent economic pests. Although the mutation has already been identified in a range of other invertebrates and vertebrates^26^, the phenotypic consequences are yet to be quantified. If consistency of effect is demonstrated, and if population suppression can ultimately be achieved, the potential applicability of this mutation is widespread. This is especially so given the rapid progress being made in gene editing technologies based on CRISPR/Cas9^35^, suggesting that it could soon be technically feasible to directly place it into target pest species rather than needing to screen for its natural occurrence.

In summary, we have provided empirical support for the TFT, comprising consistent reductions in male fertility linked to a candidate mutation in the *mt:Cyt-b* gene of the BRO haplotype of the fruit fly. Mathematical modelling predicts that even the lowest reduction in male fertility observed in the current study (*i.e.* a 35% reduction in fertility for BRO compared to PM in the CH background) could still theoretically cause marked suppression of natural populations (up to 38%) as its frequency increases (see Supplementary Information). In the longer term, TFT development will be facilitated by the identification of other mtDNA mutations exhibiting male-biased fertility effects in natural populations. While evolutionary theory suggests such mutations should be common^17^,^18^, very few studies to date have looked for them. A next step is thus to employ approaches that screen for such mutations, with the potential for multiple mutations to be coupled, both with each other and the already identified Ala278→Thr mutation of the BRO haplotype, for enhanced male fertility reduction and resultant population suppression effects.

Ultimately, however, the feasibility of the TFT requires testing at the population level by introducing the Ala278→Thr *mt:Cyt-b* mutation into test populations of *D. melanogaster*. This is particularly warranted considering that the conceptual framework and previously-published modeling underpinning the TFT to date assumes complete sterility of affected males^36^. While our experiments here document consistently-strong male-sterilizing effects of the mutation across thermal and nuclear genomic contexts, it did not confer complete male sterility. Rather, the mutation appears to be compensated for, at least in part, by modifier alleles present within the nuclear genomic variation of the outbred lines. This limited the observed reduction in male fertility to approximately 50% of wild-type. Trials in which laboratory populations are seeded with varying proportions of females harboring the BRO haplotype would thus provide both a first test for numerical suppression at a population scale, and insights into the maintenance of the haplotype across generations. If successful, we note that the development of the TFT could have profound benefits that extend far beyond those of pest control, to the control of vectors that carry human diseases such as Dengue and Zika virus.

## Methods

### Fly strains

The experiment utilised three naturally-occurring mitochondrial haplotypes, sourced from Brownsville USA [BRO], Puerto Montt Chile [PUE], and Zimbabwe [ZIM]^25^. Each of these haplotypes had been placed alongside an isogenic nuclear background *w*^1118^ 22, creating distinct “mitochondrial strains”. The BRO haplotype harbours the candidate TFT mutation (Ala278→Thr) in the mitochondrial cytochrome b gene (*mt:Cyt-b*), conferring complete male sterility but normal female fertility when expressed alongside the *w*^*1118*^ background^26^. Both sexes of the PUE and ZIM haplotypes are putatively healthy^27^,^36^; these haplotypes thus acted as controls. All mitochondrial strains have been maintained in independent duplicates since 2007 (hereafter “mitochondrial strain duplicates”), which were thereafter propagated as separate entities, with virgin daughters of each strain duplicate back-crossed to males of the *w*^*1118*^ strain for a further 60 generations to ensure that all four chromosomes in the *w*^*1118*^ nuclear background were isogenic across all mitochondrial strains. The *w*^*1118*^ strain itself is propagated by one pair of full-siblings. Each generation, virgin females of each mitochondrial strain duplicate are backcrossed to males of *w*^*1118*^, thus maintaining nuclear isogenicity across strains. Nuclear isogenicity across mitochondrial strains, coupled with the use of independent duplicates per strain, allowed us to partition true mitochondrial effects from nuclear effects and other sources of variance.

We also created a strain of flies that generated ‘tester’ females of standardized genotype for the male fertility assays. This strain was created by non-reciprocally crossing two near-isogenic lines together, each derived from the same laboratory population^37^, to create F_1_ females that exhibited high genome-wide heterozygosity. All vials were density-controlled throughout the experiment, at 10 pairs of adults and then 80 eggs per vial following ovipositioning, and maintained under constant environmental conditions. All crosses described involved flies of standard-age (3 days), and maintained on a potato-dextrose-agar medium at constant temperature (25.0 ± 0.1 °C), and diurnal cycle (12 h:12 h light:dark).

### Experimental design

Fly strains were reared at each of three temperatures (18 °C, 22 °C, and 27 °C) for two generations prior to the assay of male fertility (*i.e.* the grandparents and parents of all flies used in the experiment were reared at the appropriate temperature). The chosen temperature range reflects thermal conditions under which *D. melanogaster* maintains complete adult fertility^38^. For each of the three temperature treatments, virgin females of each mitochondrial strain duplicate were crossed to males from each of three distinct outbred laboratory populations: Coffs Harbour Australia [CH]^39^, Dahomey Benin [DAH],^40^, and California USA [LHM]^37^, (Table S1, Fig. S1). Three outbred nuclear lines from three different continents were chosen to maximise the nuclear allelic variation that mitochondrial haplotypes were expressed against, with the aim of testing the robustness of male-sterilizing effects and thus the general utility of the candidate TFT mutation. The F_1_ sons, each of whom possessed one of the three mitochondrial haplotypes (BRO, PUE, or ZIM), a haploid nuclear copy of the *w*^*1118*^ strain (inherited from the mother), and a paternally-contributed haploid nuclear copy derived from one of the three laboratory populations (CH, DAH, or LHM), were collected as virgins and used as the focal males in the fertility assays (Table S1, Fig. S1).

### Fertility assay

For each of the three temperature treatments, each focal male was maintained with two virgin ‘tester’ females for 24 h. Males were then removed, and females left to oviposit for another 24 h. Each pair of females was then transferred to a new vial every 24 h, for another two days, enabling further ovipositing. Male fertility was scored as the number of offspring eclosing from the four vials associated with each focal male. We also counted the number of pupae that did not result in adult eclosion, and were thus able to estimate pupal-to-adult viability across mtDNA haplotypes, nuclear backgrounds and temperatures.

### Statistical analysis

The structure of the data is outlined in Fig. S1. We fitted general linear mixed models to the fertility data. We also fitted generalized linear mixed models to the pupal viability data, with a binomial distribution, using the lme4 package^41^ in *R* 3.0.3^42^. The response variable was a binomial vector consisting of the number of pupae that resulted in viable offspring eclosing, and the number of pupae that did not result in viable eclosion, per male. For both analyses we treated mtDNA haplotype (BRO, PUE, ZIM), nuclear background (CH, DAH, LHM), and temperature as fixed effects, and mitochondrial strain duplicate and the vial identity in which the focal males were reared from egg to adults, as random effects. Final models of fixed effects were deduced by sequentially removing terms that did not change the deviance of the model; starting with the highest-order interactions. Log-likelihood ratio tests (and an α of 0.05) were used to assess the change in deviance in the reduced model relative to the previous model. Significance of fixed effects in the final model was then assessed using Type III sums-of-squares, χ^2^ tests, and maximum likelihood estimation in the *car* package^43^. Random effect estimates were calculated using REML.

## Additional Information

**How to cite this article**: Wolff, J. N. *et al*. Mitonuclear interactions, mtDNA-mediated thermal plasticity, and implications for the Trojan Female Technique for pest control. *Sci. Rep.* **6**, 30016; doi: 10.1038/srep30016 (2016).

## Supplementary Material

Supplementary Information

Supplementary Information

## Figures and Tables

**Table 1:** Sources of variance affecting male reproductive success.

***Fixed effects***	**χ**^**2**^	**df**	**p**
**Source**			
mtDNA haplotype	8.94	2	0.0114
Nuclear background	45.13	2	<0.001
Temperature	32.76	2	<0.001
mtDNA × nuclear	36.56	4	<0.001
mtDNA × temperature	24.90	4	<0.001
*Random effects*	**Standard deviation**		
Vial ID	26.29		
mtDNA duplicate	0		
Residual	64.59		
