# Supplementary material for: Mitonuclear interactions, mtDNA-mediated thermal plasticity, and implications for the Trojan Female Technique for pest control
Source: Sci Rep. 2016 Jul 21;6:30016. doi: 10.1038/srep30016 (PMC4956753; doi:10.1038/srep30016)
Supplement: Supplementary Information [file srep30016-s1.pdf]

Mitonuclear interactions, mtDNA-mediated thermal plasticity, and implications for the Trojan Female Technique for pest control

Jonci N. Wolff[1,*], Daniel M. Tompkins[2], Neil J. Gemmell[3], Damian K Dowling[1]

[1] School of Biological Sciences, Monash University, Victoria, 3800, Australia

[2] Landcare Research, Private Bag 1930, Dunedin, New Zealand

[3] Allan Wilson Centre for Molecular Ecology and Evolution, Department of Anatomy, University of Otago, Dunedin 9016, New Zealand

[*]Corresponding author

Supplementary Material

Supplementary Tables

Table S1: Experimental breeding scheme. Crosses were established between females of three duplicated mitochondrial lines (Brownsville [BRO], Puerto Montt [PUE], Zimbabwe [ZIM]), and males of three distinct outbred fly strains (Coffs Harbour [CH], Dahomey [DAH], LH_M [LHM]). The BRO haplotype is known to confer reduced male fertility, and this is traceable to a candidate TFT mutation in the *mtCYTB* gene; PUE and ZIM haplotypes served as control haplotypes. All nine crosses were independently replicated, enabled through the maintenance of two independent duplicates per mitochondrial line since their inception in 2007. This breeding scheme generated focal males for our fertility assays that possessed one of the three mitochondrial haplotypes, a haploid copy of the w^{1118} nuclear background (inherited from the mother), and a haploid nuclear copy of one of the three outbred nuclear backgrounds (inherited from the father).

**Maternal contribution**		**Paternal contribution**		
Mitochondrial lines		Coffs Harbour (CH)	Dahomey (DAH)	LH_M (LHM)
TFT	BRO 1	BRO 1 mtDNA w^{1118}:CH	BRO 1 mtDNA w^{1118}:DAH	BRO 1 mtDNA w^{1118}:LHM
	BRO 2	BRO 2 mtDNA w^{1118}:CH	BRO 2 mtDNA w^{1118}:DAH	BRO 2 mtDNA w^{1118}:LHM
Control 1	PUE 1	PUE 1 mtDNA w^{1118}:CH	PUE 1 mtDNA w^{1118}:DAH	PUE 1 mtDNA w^{1118}:LHM
	PUE 2	PUE 2 mtDNA w^{1118}:CH	PUE 2 mtDNA w^{1118}:DAH	PUE 2 mtDNA w^{1118}:LHM
Control 2	ZIM 1	ZIM 1 mtDNA w^{1118}:CH	ZIM 1 mtDNA w^{1118}:DAH	ZIM 1 mtDNA w^{1118}:LHM
	ZIM 2	ZIM 2 mtDNA w^{1118}:CH	ZIM 2 mtDNA w^{1118}:DAH	ZIM 2 mtDNA w^{1118}:LHM

Table S2. Sources of variance affecting pupal viability.

Fixed effects

**Source**	**χ^2**	**df**	**p**
mtDNA haplotype	15.34	2	0.0004
Nuclear background	9.07	2	0.0107
Temperature	10.71	2	<0.0001
mtDNA × nuclear	17.36	4	0.0011
nuclear × temperature	37.66	4	<0.001
Random effects	**Standard deviation**		
Male ID	5.22×10^{-1}		
Vial ID	2.49×10^{-6}		
mtDNA duplicate	0		

Supplementary Figures

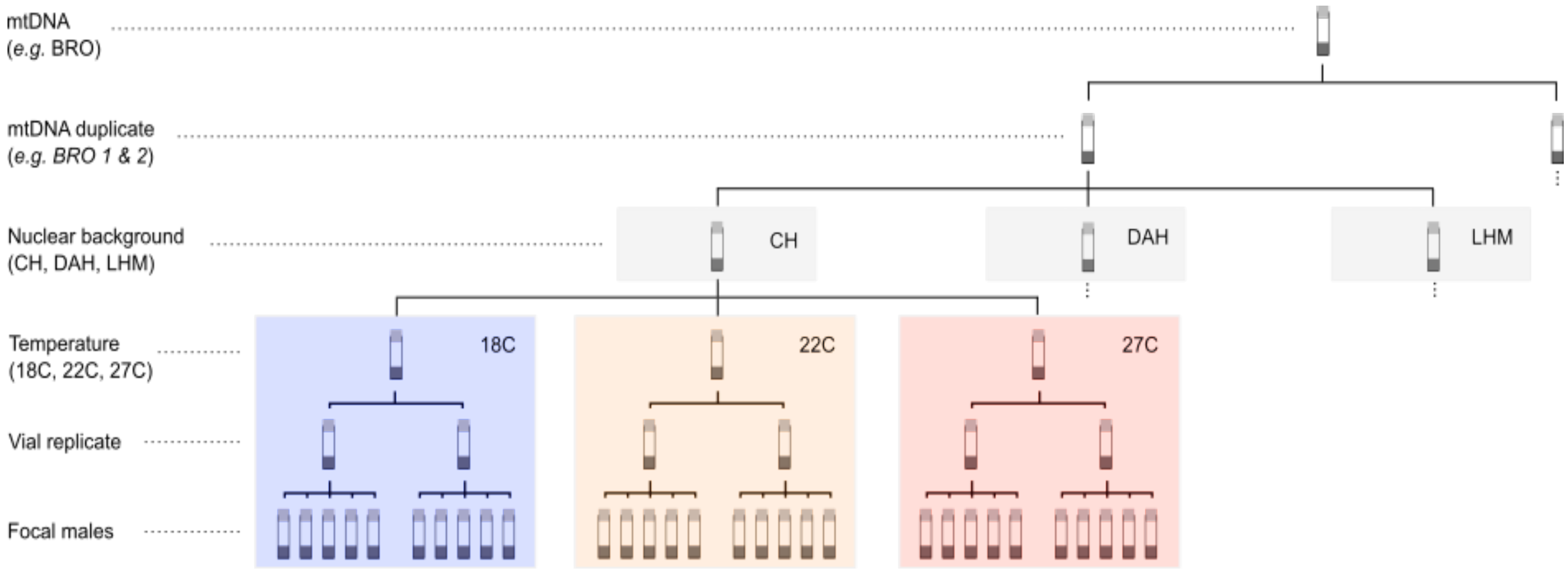

Figure S1: Experimental breeding scheme. Mitochondrial strains (Brownsville [BRO], Puerto Montt [PUE], Zimbabwe [ZIM]) were maintained in independent duplicates since 2007 (*e.g.* BRO 1 & 2). To generate focal males, mitochondrial strains were maintained at three different temperatures (18°C, 22°C, 27°C). Virgin females of each mitochondrial strain duplicate were crossed to males of three nuclear backgrounds (Coffs Harbour [CH], Dahomey [DAH], LH_M [LHM]). The three mitonuclear lineages were replicated over two vials, and five focal males sampled per replicate vial.
