# Supplementary material for: Mitonuclear interactions, mtDNA-mediated thermal plasticity, and implications for the Trojan Female Technique for pest control
Source: Sci Rep. 2016 Jul 21;6:30016. doi: 10.1038/srep30016 (PMC4956753; doi:10.1038/srep30016)
Supplement: Supplementary Information [file srep30016-s2.doc]

Mitonuclear interactions, mtDNA-mediated thermal plasticity, and implications for the Trojan Female Technique for pest control

Jonci N. Wolff^1,*^, Daniel M. Tompkins^2^, Neil J. Gemmell^3^, Damian K Dowling^1^

^1^ School of Biological Sciences, Monash University, Victoria, 3800, Australia

^2^ Landcare Research, Private Bag 1930, Dunedin, New Zealand

^3^ Allan Wilson Centre for Molecular Ecology and Evolution, Department of Anatomy, University of Otago, Dunedin 9016, New Zealand

^*^Corresponding author

Appendix

Predicted impacts of the Trojan Female Technique on *Drosophila melanogaster* populations

**Summary**

- A simulation model of laboratory Drosophila melanogaster populations was constructed by adapting an applicable existing model from Rodriguez (1989). The adapted model includes the capacity to simulate both TFT and wildtype mtDNA haplotypes, the capacity to introduce individuals containing the TFT haplotype with varying magnitude of effect on male fertility, the capacity to vary the level of female re-mating, stochastic genetic drift of haplotypes over time, and stochasticity in life-history parameters.
- For laboratory *D. melanogaster* population simulations, in which female mating with multiple males each generation was allowed, clear demonstrable effects of TFT haplotypes were predicted. Model exploration demonstrated that the size of population reduction achieved and maintained by the TFT (i.e. population suppression) in laboratory populations is predicted to increase with both greater sterilising effects, and higher frequency, of the TFT haplotype.
- When the *D. melanogaster* mating dynamics simulated were adjusted to better reflect the situation in natural populations (i.e. mostly single mating each generation), it was shown that the sterilising effect / haplotype frequency combinations resulting in relatively low predicted suppression of laboratory populations could theoretically have much greater impact. For example, the only 24% suppression predicted to be caused by a TFT haplotype causing complete male sterility at a frequency of 0.6 in laboratory populations increased to 75% under the more natural breeding dynamics.

**Introduction**

A new approach to pest fertility control has been proposed, called the ‘Trojan Female Technique’ (TFT). The TFT is a novel twist on the successful Sterile Insect Technique (SIT) paradigm. Rather than releasing large quantities of sterile males on a yearly basis (as in the SIT, with the sterile males normally created through irradiation), the TFT involves the release of females carrying naturally occurring mitochondrial DNA haplotypes that reduce the fertility of their male offspring (Gemmell et al. 2013).

Development of the TFT is being carried out with *Drosophila melanogaster* (Dowling *et al*. 2015). Here a non-spatial mathematical model is constructed for this species to simulate laboratory population suppression trials and predict the impact that observed effects would have on natural populations. The population suppression achievable by the TFT is expected to be highly dependent on the number of males that females mate with (Gemmell *et al*. 2013), with this rate known to vary greatly between laboratory and wild *D. melanogaster* (Griffiths et al. 1982; Gromko & Gerhart 1984). Since there is no sufficiently parameterised model available for natural *D. melanogaster* populations, we thus explored how any suppressive effect predicted for laboratory populations would translate across for more natural situations by altering this rate in the model.

Model construction

The underlying model used is the stochastic model developed by Rodriguez (1989), constructed to simulate laboratory subpopulations of *D. melanogaster* maintained in a standard serial-passage format. The model is parameterised from, and simulates, subpopulations maintained as discrete generations in 8-dram (35 ml) vials with 10 ml of food. The model explicitly includes density-dependence effects of pre-adult density on both pre-adult survival and adult fecundity, and adult density on adult fecundity. By using this model, TFT simulations will thus encompass any release from density-dependence that might buffer populations from reduced male fertility effects. The model allows for stochasticity in pre-adult survival, adult fecundity and adult sex ratio, with all parameter values and levels of variation based on empirical data gathered from the subpopulations. The incorporation of such stochasticity is necessary for the realistic simulation of drosophila populations. The Rodriguez (1989) stochastic model can be represented by:


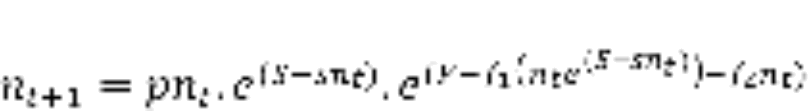


This is a ‘recursion’ equation giving the number of eggs output per generation (
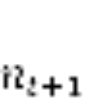
) as a function of number of eggs input (
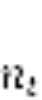
). The component
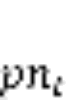
 calculates the number of female eggs (*p* is the proportion of females). The component
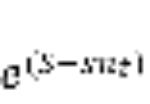
 calculates the sub-adult survival rate, where *S* relates to density-independent survival, and *s* relates to density-dependent reductions in survival. The component
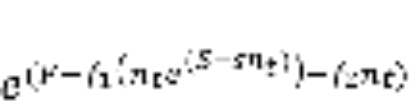
 calculates the female fecundity rate, where *F* relates to density-independent fecundity,
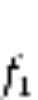
 relates to the density-dependent effect of adult density on adult fecundity, and
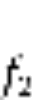
 relates to the density-dependent effect of pre-adult density on adult fecundity.

To incorporate the effects of an mtDNA haplotype reducing male fertility (with no other effects), we let *x* represent the proportion of the subpopulation that carries the wildtype mtDNA, with all others carrying the reduced male fertility haplotype (the ‘TFT’ haplotype). The probability of an adult female receiving wildtype male sperm is determined by this frequency and the number of males with which each female mates. While *D. melanogaster* females re-mate at a relatively low frequency in natural populations, with the average number of mates rapidly closing on one as population density decreases (Marks et al. 1988), in continuous laboratory populations the re-mating of close to all females can occur (with the number of males each female mates with unknown; Gromko and Gerhart 1984). In addition, sperm depletion from initial mating’s is known to trigger female *D. melanogaster* re-mating at laboratory population densities (Gromko & Pyle 1978); mating with a TFT male with impaired fertility would likely have similar effects. Our model is thus constructed to allow simulation of different mean rates of female re-mating, by calculating the ‘effective’ frequency of the wildtype mtDNA haplotype (
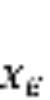
) as
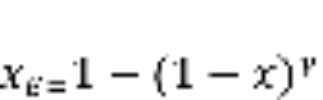
, where *y* is the average number of males that each female mates with for each generation. This incorporation of multiple mating assumes that mating with a single wildtype male is sufficient for all TFT effects to be negated. If
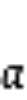
 represents the fertility of TFT haplotype males (as a proportion of wildtype fertility), the fertility rate of eggs input into each generation (i.e. the proportion of the input eggs that are fertile) is calculated as
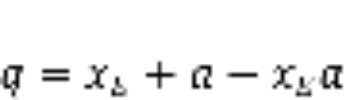
. This term can then be included into the recursion equation such that egg output per generation as a function of egg input is influenced by the proportion that are fertile:


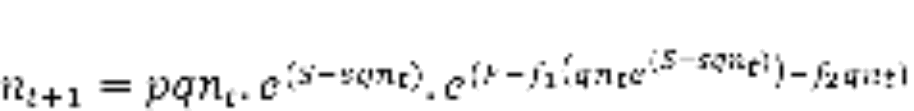


Model parameterisation


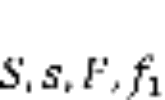
 and
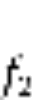
 are considered as normally distributed random variables with distribution parameters calculated as in Rodriguez (1989) and listed in Table A1. The number of female fertile eggs input (
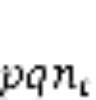
) is generated by multiplying *p* by a random variable with binomial distribution approximated by a normal with a mean of
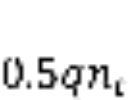
 and a standard deviation of
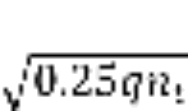
 (Rodriguez 1989).

In addition to this stochasticity in pre-adult survival, adult fecundity and adult sex ratio, we consider the frequency of the wildtype mtDNA haplotype (
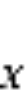
) to be susceptible to genetic drift. Birky et al. (1983) show how for sexually reproducing individuals in which organelles are homoplasmic and inheritance is solely maternal, the variance of the change in frequency of an mtDNA haplotype caused by stochastic sampling of a population from one generation to the next can be approximated by
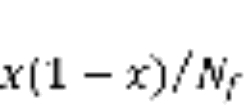
, where
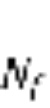
 is the number of adult females (in our case, the number of adult females that would have laid the input eggs). The wildtype frequency for each successive generation is thus randomly sampled from a normal distribution with a mean equating to the frequency in the previous generation, and this variance (calculated from the previous generation), with lower and upper limits of 0 and 1, respectively (at which point the wildtype haplotype is either lost from the population or fixed in the population, and no further drift occurs).

Table A1 Mean and standard deviation for the normally distributed random variables included in the *Drosophila melanogaster* laboratory population model

| Parameter | Mean | Standard deviation |
| --- | --- | --- |
| 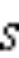 | -0.510581 | 0.037306 |
| 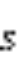 | 0.001335 | 0.000060 |
| 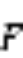 | 2.28345 | 0.303361 |
| 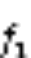 | 0.0112493 | 0.003278 |
| 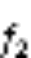 | 0.000855 | 0.000114 |
| 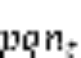 | 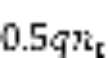 | 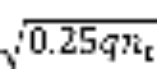 |
| 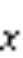 | 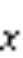 | 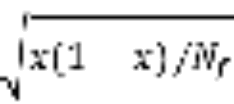 |

Model simulation

The non-stochastic version of the Rodriguez (1989) model has an equilibrium population size of approximately 140 eggs output per generation. Thus, we initiate all model iterations with
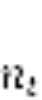
 = 140 eggs, and a corresponding initial value for
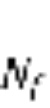
 of 35 (calculated from Eqn 8 in Rodriguez 1989). Each model iteration is run for 10 generations, with 1000 iterations per scenario, and fly population size in the tenth generation of the 1000 iterations reported on for each scenario. Thirty-six different scenarios are simulated: all combinations of initial
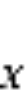
 (wildtype mtDNA frequency in the initial input eggs) varied from 1 to 0 in steps of -0.2, and
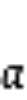
 (the fertility of TFT haplotype males as a proportion of wildtype fertility), likewise varied from 1 to 0 in steps of -0.2.

The full set of simulations is run twice, once under the assumption that females are mating on average with three males each generation (i.e. *y* = 3), reflecting laboratory mating dynamics, and once with single mating only each generation (i.e. *y* = 1), allowing an assessment of predicted suppression if mating dynamics were more representative of natural populations.

Results

As in Rodriguez *et al.* (1989), the model produced highly variable fruit fly population dynamics, very similar to those observed in laboratory populations. Wildtype fruit fly populations (i.e. with no TFT individuals) fluctuated haphazardly with adult densities generally occurring between 50 –100 per vial. Despite the variation observed in the baseline dynamics, clear suppressive effects of TFT haplotypes on laboratory *D. melanogaster* populations were predicted (Figure A1a). The predicted size of laboratory population reduction achieved and maintained by the TFT (i.e. population suppression) increases with both greater sterilising effects, and higher frequency, of the TFT haplotype. Effects of genetic drift on TFT haplotype frequency were negligible over the 10-generation model runs.

Although the predicted suppression of laboratory populations rapidly decreased as either the TFT haplotype frequency or its sterilising effect decreased, altering the modelled breeding dynamics to better reflect those which occur in natural populations dramatically ameliorated the loss of suppressive effect (Figure A1b). For example, the only 24% population suppression predicted to be caused by a TFT haplotype causing complete male sterility at a frequency of 0.6 in laboratory populations increased to 75% under the more natural breeding dynamics. This illustrates the expected effect of female *D. melanogaster* re-mating under laboratory conditions in reducing population suppression caused by the TFT.

Reducing model density dependence parameters (while keeping their relative strengths constant), to result in larger populations being simulated (i.e. again more representative of natural dynamics), did not lead to any change in the relative size of suppressive effects predicted (results not shown). A future informative step would be to extend to spatial modelling of population dynamics, since non-panmictic mixing in natural populations, and immigration and emigration patterns, could all influence predicted suppressive effects. However, such models (to be adapted for our purpose) are not yet available in the literature, with lack of necessary field data for parameterisation likely being a key hurdle.

**Figure A1** Predicted sizes of *Drosophila melanogaster* laboratory populations (y-axis) relative to TFT haplotype frequency (x-axis) and the fertility of TFT haplotype males as a proportion of wildtype fertility (legend). Simulations were run for females mating (a) on average with three males each generation, reflecting laboratory dynamics, and (b) only once each generation, being more representative of natural populations.

(a) (b)


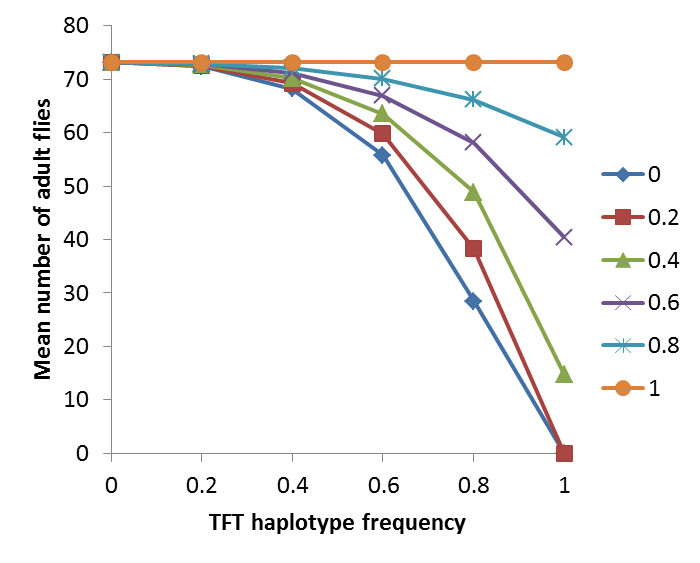

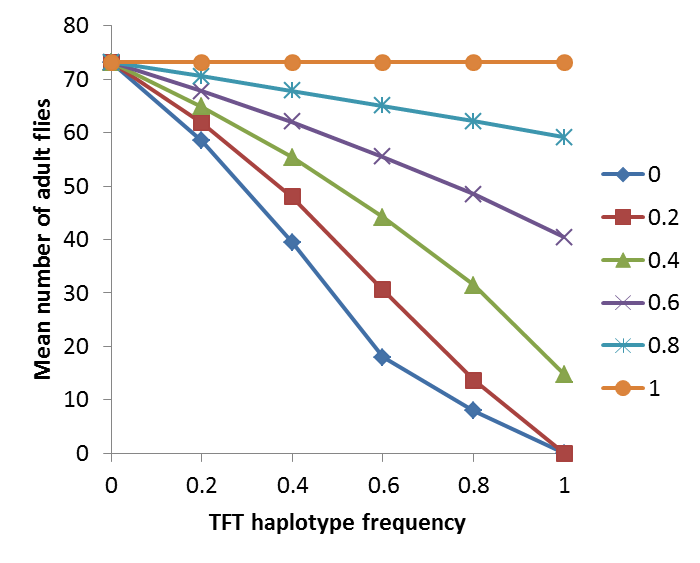


References

Birky CW, Maruyama T, Fuerst P (1983) An approach to population and evolutionary genetic theory for genes in mitochondria and chloroplasts. *Genetics* 103: 513-527.

Dowling DK, Gemmell NJ, Tompkins DM (2015) The Trojan Female Technique for pest control: a candidate mitochondrial mutation confers low male fertility across diverse nuclear backgrounds and mating contexts in *Drosophila melanogaster*. *Evolutionary Applications* doi:10.1111/eva.12297

Gemmell NJ, Jalilzadeh A, Didham RK, Soboleva T, Tompkins DM (2013) The Trojan female technique: a novel, effective and humane approach for pest population control. *Proceedings of the Royal Society B - Biological Sciences* 280: 20132549.

Griffiths RC, McKechnie SW, McKenzie JA (1982) Multiple mating and sperm displacement in a natural population of *Drosophila melanogaster*. *Theoretical and Applied Genetics* 62: 89-96.

Gromko MH, Gerhart PD (1984) Increased density does not increase remating frequency in laboratory populations of *Drosophila melanogaster*. *Evolution* 38(2): 451-455.

Gromko MH, Pyle DW (1978) Sperm competition, male fitness, and repeated mating by female *Drosophila melanogaster*. *Evolution* 32: 588-593.

Marks RW, Seager RD, Barr LG (1988) Local ecology and multiple mating in a natural population of *Drosophila melanogaster*. *American Naturalist* 131(6): 918-923.

Rodriguez DJ (1989) A model of population dynamics for the fruit fly *Drosophila* *melanogaster* with density dependence in more than one life stage and delayed density effects. *Journal of Animal Ecology* 58: 349-365.
